# Supplementary material for: Multi-tissue analyses of allele-specific chromatin accessibility nominate likely functional variants for type 2 diabetes
Source: medRxiv. 2026 Jul 15:2026.07.14.26358094. Preprint. [Version 1] doi: 10.64898/2026.07.14.26358094 (PMC13405542; doi:10.64898/2026.07.14.26358094)

Supplementary figure 2

A ASAI SNP rs13051142 was missed by the caQTL due to a moderate effect size. The same SNP is an eQTL for *LCA5L* in the same Type 1 muscle cells

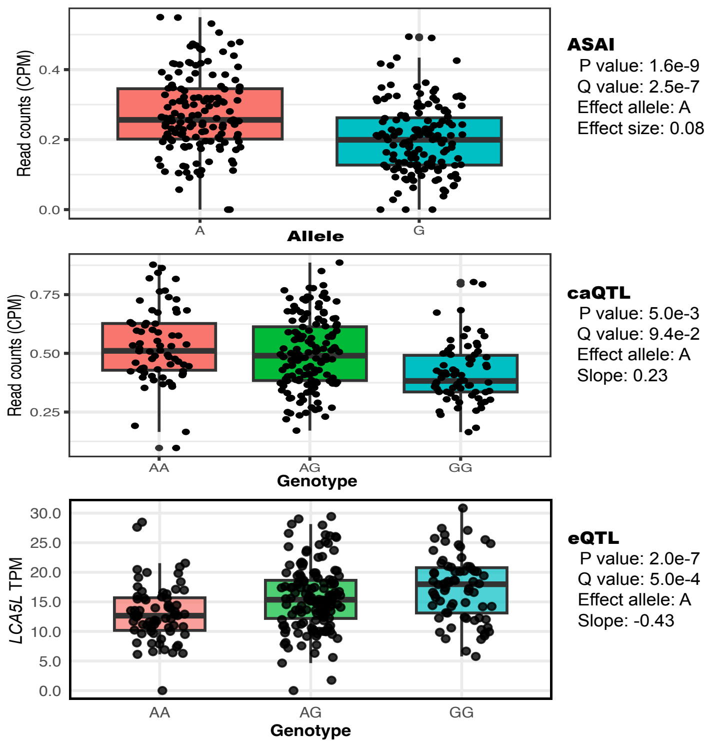

B caQTl SNPs missed by ASAI tend to have lower ATAC sequencing coverage and lower MAF in Type 1 muscle cells

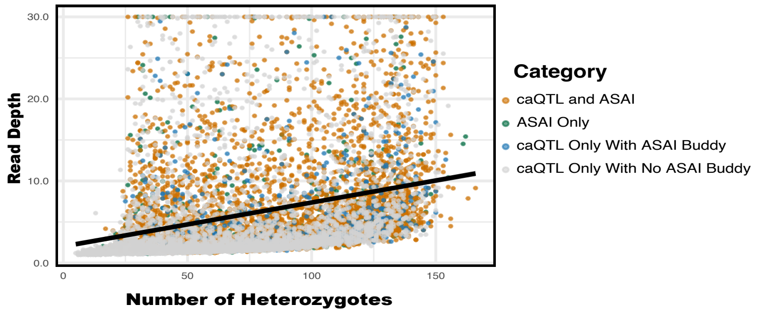

C Lead caQTL SNPs missed by ASAI tend to have lower MAF in Type 1 muscle cells

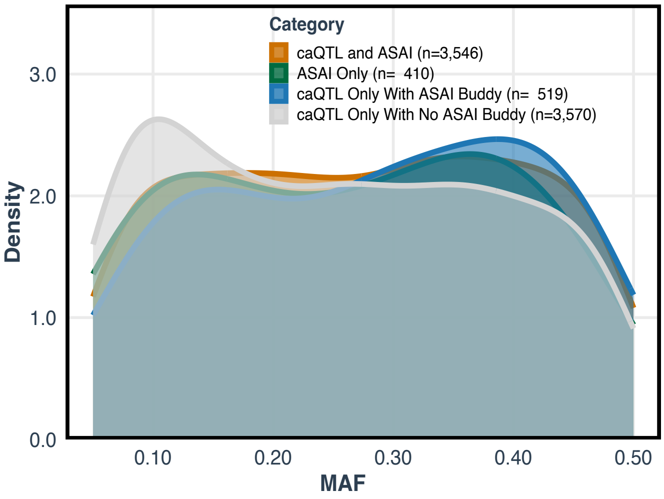

Supplement: Supplementary file 1 [file media-1.zip › supplemental_materials/Supplementary_Figure2_03_27_2026.pdf]
