# Supplementary material for: Multi-tissue analyses of allele-specific chromatin accessibility nominate likely functional variants for type 2 diabetes
Source: medRxiv. 2026 Jul 15:2026.07.14.26358094. Preprint. [Version 1] doi: 10.64898/2026.07.14.26358094 (PMC13405542; doi:10.64898/2026.07.14.26358094)

Supplementary figure 3

A Top 25 TFBSs enriched to overlap with allelic imbalanced SNPs in muscle pseudo bulk cells

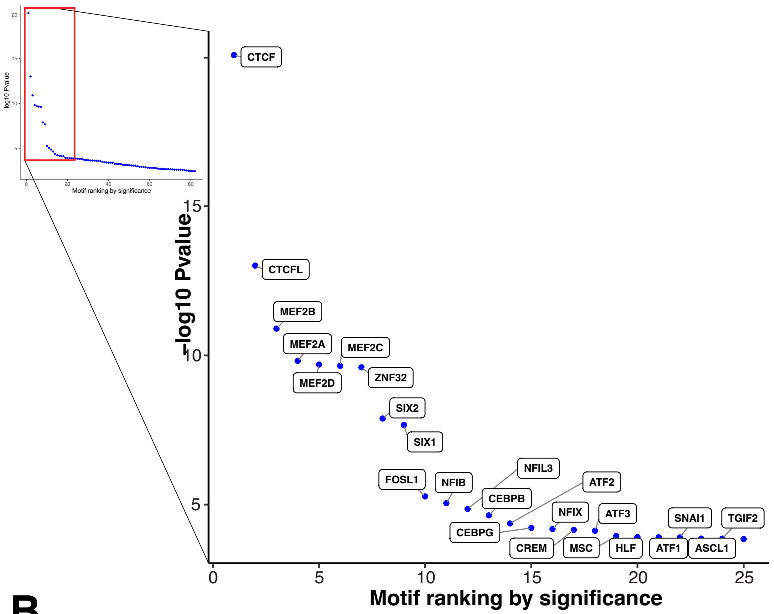

B Top 25 TFBSs enriched to overlap with allelic imbalanced SNPs in liver cells

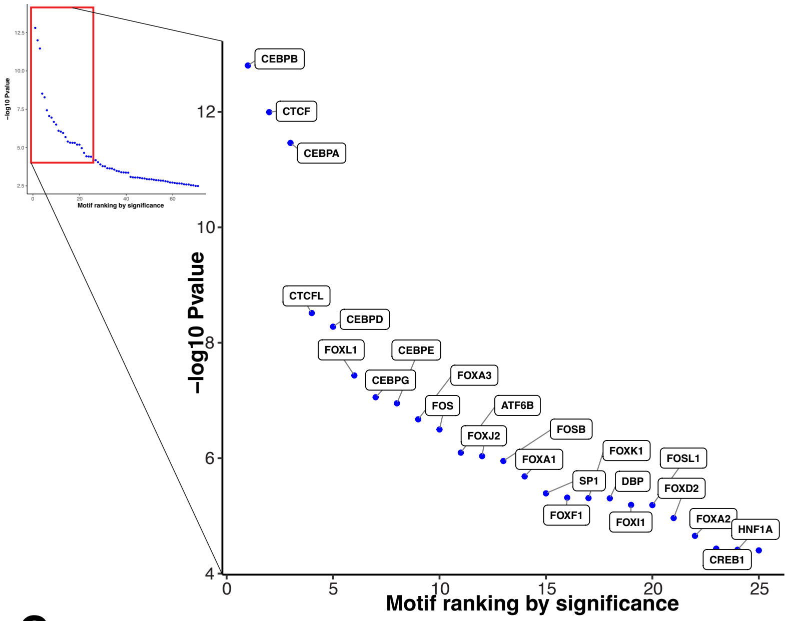

C Top 25 TFBSs enriched to overlap with allelic imbalanced SNPs in islet cells

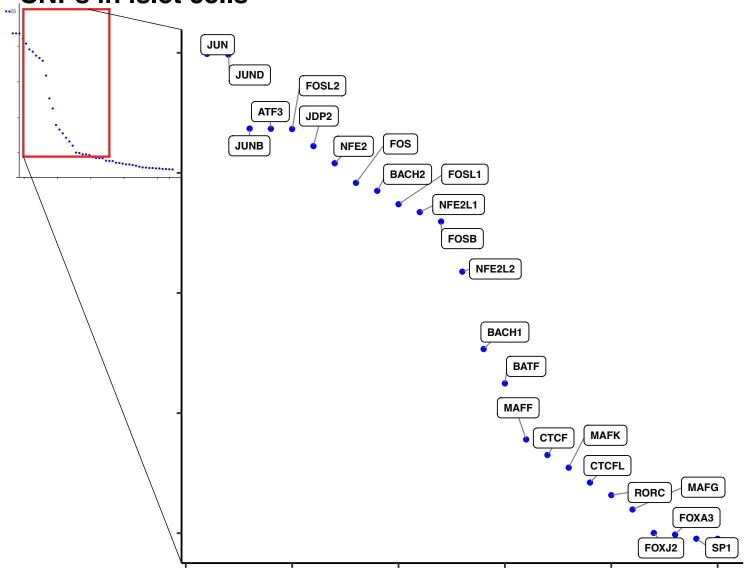

D TFBSs enriched to overlap allelic imbalanced SNPs tend to be tissue specific

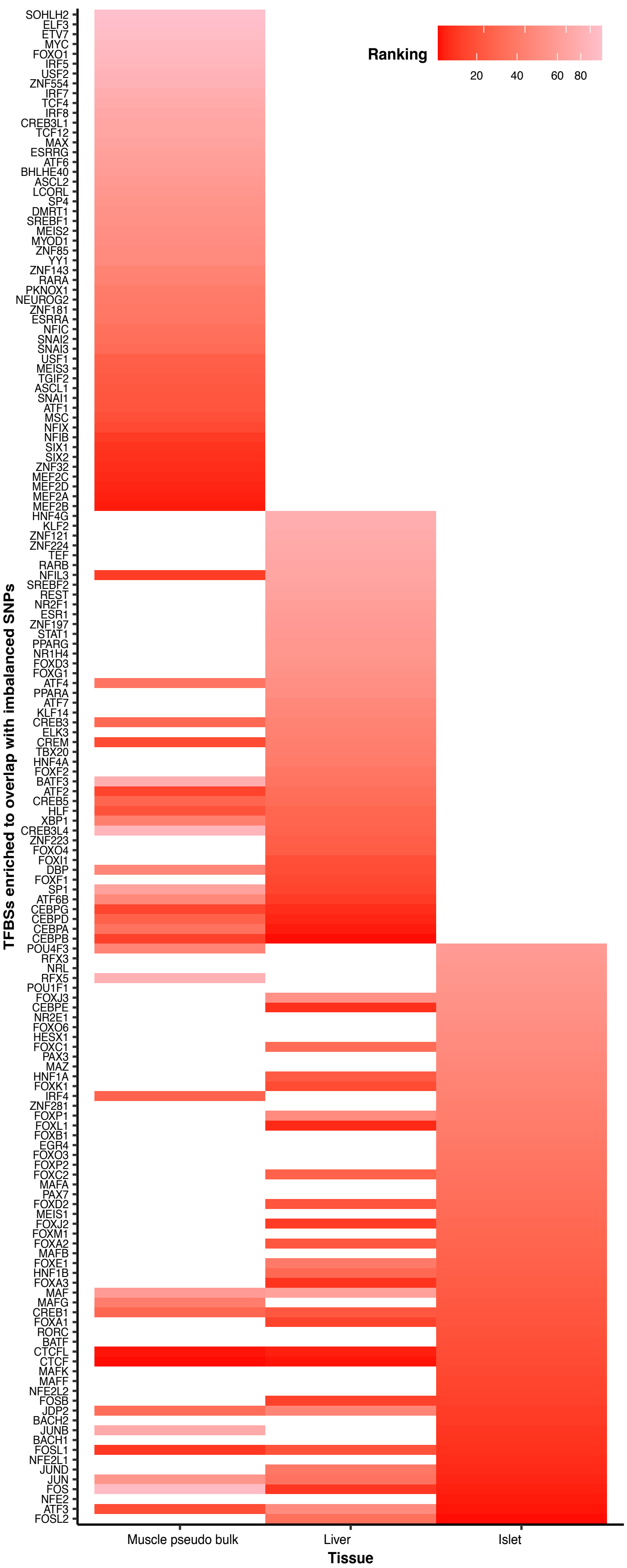

Supplement: Supplementary file 1 [file media-1.zip › supplemental_materials/Supplementary_Figure3_09_08_2025.pdf]
