# Supplementary material for: Multi-tissue analyses of allele-specific chromatin accessibility nominate likely functional variants for type 2 diabetes
Source: medRxiv. 2026 Jul 15:2026.07.14.26358094. Preprint. [Version 1] doi: 10.64898/2026.07.14.26358094 (PMC13405542; doi:10.64898/2026.07.14.26358094)

Supplementary figure 5

A Previously identified T2D causal SNP rs7163757 at C2CD4A/B signal in skeletal muscle cells

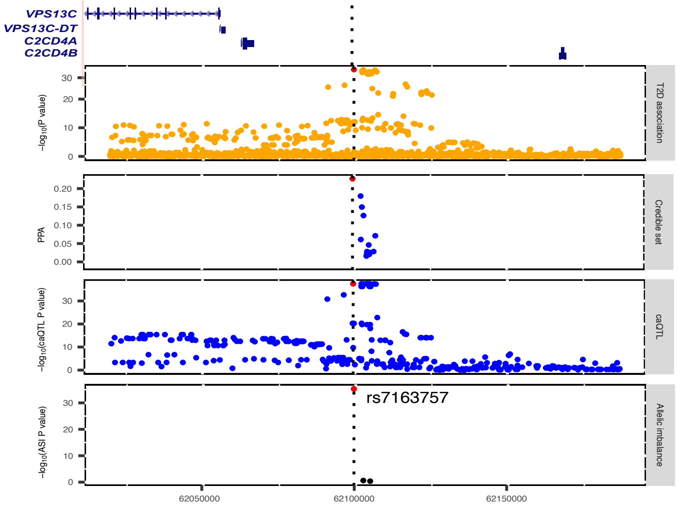

B Allelic imbalanced T2D credible SNP rs34584161 at RNF6 signal

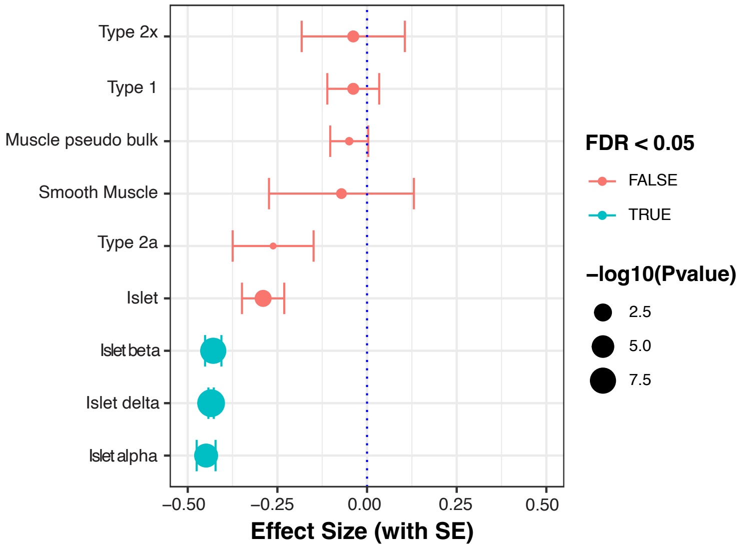

C Allelic imbalanced candidate T2D SNP rs34584161 at RNF6 signal overlaps an ATAC peak in islet cell types

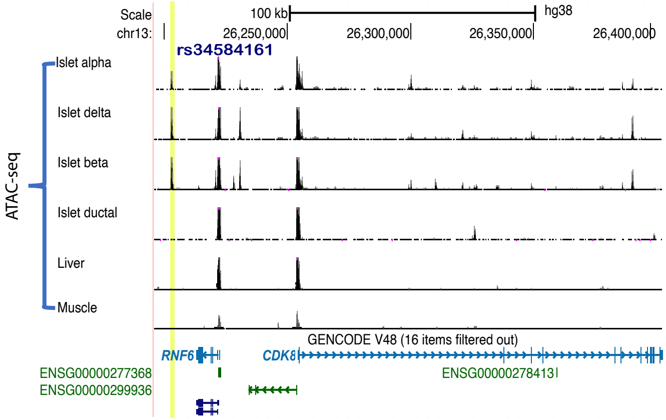

D ATAC-seq peak overlapping candidate T2D causal SNP rs849134 at JAZF1 signal in liver cells

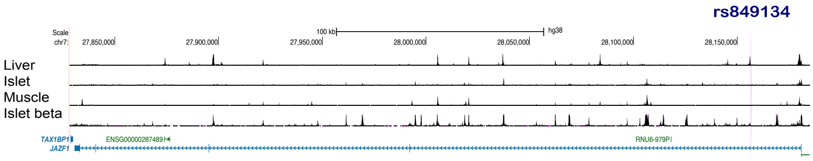

E EMSA and relative luciferase reporter activity of rs849135 at JAZF1 signal in HepG2 cells

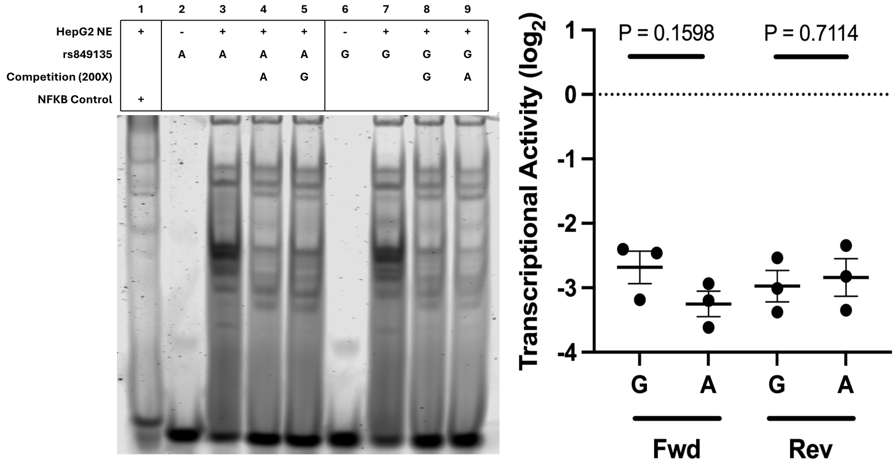

F Candidate T2D causal SNP rs5398 at SLC2A2 signal in liver cells

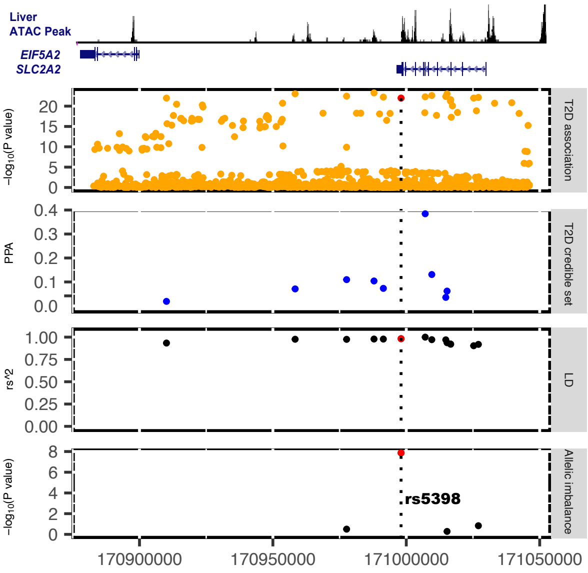

Supplement: Supplementary file 1 [file media-1.zip › supplemental_materials/Supplementary_Figure5_05_22_2026.pdf]
